# The interplay between the microbiota and opioid in the treatment of neuropathic pain

**DOI:** 10.3389/fmicb.2024.1390046

**Published:** 2024-06-10

**Authors:** Zexiong Gong, Qingsheng Xue, Yan Luo, Buwei Yu, Bo Hua, Zhiheng Liu

**Affiliations:** ^1^Department of Anesthesiology, Health Science Center, Shenzhen Second People’s Hospital/The First Affiliated Hospital of Shenzhen University, Shenzhen, China; ^2^Department of Anesthesiology, School of Medicine, Ruijin Hospital, Shanghai Jiaotong University, Shanghai, China

**Keywords:** gut microbiota, gut-brain axis, neuropathic pain, opioids, pain

## Abstract

Neuropathic pain (NP) is characterized by its complex and multifactorial nature and limited responses to opioid therapy; NP is associated with risks of drug resistance, addiction, difficulty in treatment cessation, and psychological disorders. Emerging research on gut microbiota and their metabolites has demonstrated their effectiveness in alleviating NP and augmenting opioid-based pain management, concurrently mitigating the adverse effects of opioids. This review addresses the following key points: (1) the current advances in gut microbiota research and the challenges in using opioids to treat NP, (2) the reciprocal effects and benefits of gut microbiota on NP, and (3) the interaction between opioids with gut microbiota, as well as the benefits of gut microbiota in opioid-based treatment of NP. Through various intricate mechanisms, gut microbiota influences the onset and progression of NP, ultimately enhancing the efficacy of opioids in the management of NP. These insights pave the way for further pragmatic clinical research, ultimately enhancing the efficacy of opioid-based pain management.

## 1 Introduction

The pathological pain resulting from damage or diseases that affects the somatosensory nervous system is called neuropathic pain (NP) (Jensen et al., 2011). NP is often accompanied by neuronal damage, and accumulating experimental evidence has highlighted the involvement of various non-neuronal cells, including glial cells, regulatory T cells, and tumor cells, as pivotal contributors to the onset of pain (Fiore et al., 2023). In this article, we describe the close interplay between gut microbiota and NP and consider the therapeutic potential of utilizing gut microbiota to augment the management of NP, particularly within the context of opioid therapy.

Recent findings in epidemiological and genomic research, coupled with cellular and animal experiments, underscore the profound influence of the microbiome on human health, acting as a mediator or modifier of environmental variables. Most of these microorganisms reside in the gastrointestinal tract, where they synthesize and modify an array of metabolites and chemical compounds. Predominantly classified into the phyla Firmicutes and Bacteroidetes, these microorganisms constitute the principal gut microbiota populations (Zádori et al., 2023). They intricately participate in modulating various physiological functions within the human body, including neuroinflammatory responses and other vital physiological processes (Belkaid and Harrison, 2017; Fan and Pedersen, 2021; Fiore et al., 2023). The development of the gut microbiota system is a gradual and intricate process beginning in infancy (Schmidt et al., 2018; Cassidy-Bushrow et al., 2023; Ruan et al., 2023). Its makeup can change over time, influenced by factors such as age, dietary habits, and prolonged medication use (Minerbi et al., 2019; Fan and Pedersen, 2021). Due to the heterogeneity of external perturbations, the rate of change in the gut microbiota varies among individuals (Flores et al., 2014). Empirical evidence consistently demonstrates that the composition of gut microbiota exhibits notable stability, with the ability to return to its baseline profile after disturbances (Dogra et al., 2020). The advent of genome sequencing technologies has significantly expedited the progress of gut microbiota research (Jovel et al., 2016). In recent years, whole-genome sequencing has unveiled the association between gut microbiota and a diverse spectrum of diseases. Initially, distinct microbial profiles were identified in the progression of gastrointestinal disorders (Barko et al., 2018). Subsequently, deviations in gut microbiota composition have been identified as playing a role inconditions spanning metabolism-related disorders, tumors, cardiovascular diseases, and pain-related disorders (Minerbi and Shen, 2022). In various forms of pain, including NP, distinctive gut microbiota signatures have been observed (Guo et al., 2019; Pane et al., 2022).

For a long time, predominant approaches to treating NP have targeted the central nervous system (CNS). Opioid medications, including morphine, tramadol, and hydrocodone have emerged as the cornerstone of clinical intervention (Balanaser et al., 2023). The escalating use of opioid medications is attributable to their excellent pain-relieving effects (Xu et al., 2022). Despite their effectiveness in helping manage moderate to severe pain, opioids often exhibit suboptimal analgesic efficacy in the treatment of NP (Martínez-Navarro et al., 2019). Classic opioids such as morphine and hydrocodone have shown limited effectiveness in this context, with clinical trials reporting unsatisfactory levels of efficacy and safety (Martínez-Navarro et al., 2019). Prolonged opioid therapy frequently leads to tolerance, dependence, and gastrointestinal adverse effects, leaving a growing number of patients unable to attain satisfactory pain relief within tolerable dose ranges. Encouragingly, a growing body of literature suggests that gut microbiota can effectively mitigate the adverse effects of opioids and contribute to the treatment of NP (Fiore et al., 2023). It is critical to note the bidirectional relationship between gut microbiota and opioids in NP treatment. Gut microbiota influence the analgesic effects of opioids, while opioids, in turn, induce alterations in the gut microenvironment (Wang et al., 2018; Zádori et al., 2023). Therefore, the gut microbiota emerges as a critical target for optimizing opioid therapy in the treatment of NP.

## 2 Gut microbiota in neuropathic pain

### 2.1 The gut-brain axis

**Figure 1:**
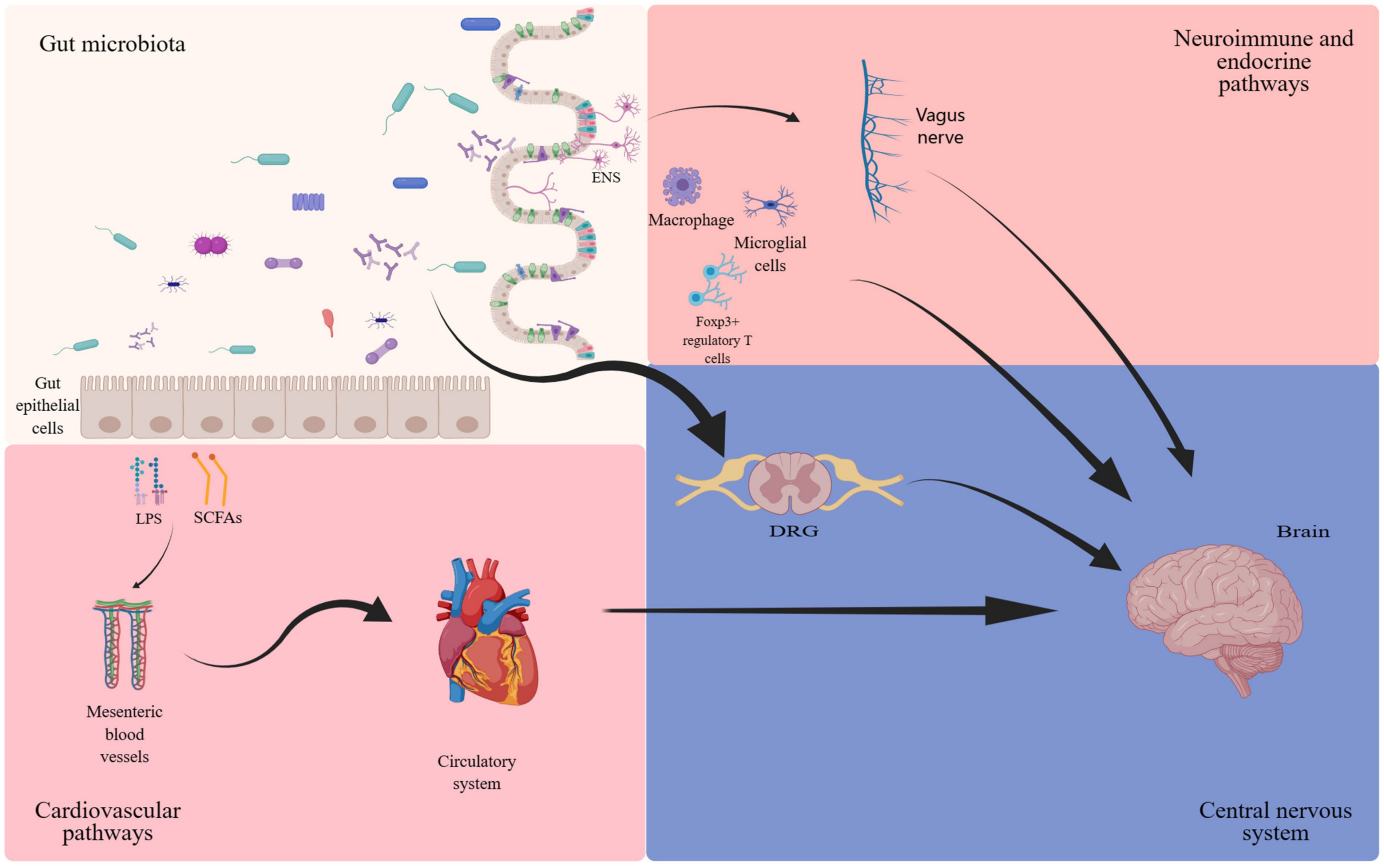
The involvement of gut microbiota in the gut-brain axis. The bidirectional communication between the gut microbiota and the brain occurs through endocrine (cortisol), immune (cytokines) and neural (vagus nerve and enteric nervous system) mechanisms (Josefowicz et al., 2012; Zhang et al., 2016; Cussotto et al., 2018; Russo et al., 2018; Guo et al., 2019; Ding et al., 2021; Dora et al., 2021; Ma et al., 2022; Araldi et al., 2023).

The gut microbiota has long been considered for its beneficial impact on intestinal motility (Dey et al., 2015), local immune function (Cai et al., 2022), and visceral pain perception (Luczynski et al., 2017; Aguilera et al., 2021). Recent evidence has further elucidated its regulatory influence on organs beyond the gastrointestinal tract, most notably its association with the nervous system (Thaiss, 2023). The concept of the gut-brain axis was first proposed in the 1880s, subsequently undergoing continuous refinements (Russo et al., 2018). The gut-brain axis constitutes a complex bidirectional communication network (Cussotto et al., 2018; Russo et al., 2018). The gut microbiota and its metabolites, such as γ- aminobutyric acid (GABA), serotonin, bile acids, and amino acids, directly influence the nervous system, and indirectly mediate cell interactions through the regulation of the vagus nerve (Figure 1). The gut-brain axis is believed to be closely linked to a range of disorders, including depression (Foster, 2022), Alzheimer’s disease (Nguyen and Endres, 2022), and autism spectrum disorders (Coccurello et al., 2022). NP, the focus of this article, has also been extensively reported to be robustly connected with the gut-brain axis. Notably, mediators derived from gut microbiota exert their effects by influencing the dorsal root ganglion (DRG) and specific T cells, thereby modulating neuronal excitability and nociception (Guo et al., 2019). Furthermore, the enteric nervous system (ENS), often referred to as the “second brain,” comprises the submucosal plexus, myenteric plexus, and interstitial cells of Cajal. It facilitates bidirectional communication with the CNS, transmitting impulses on both directions (Morreale et al., 2022). While typically under the regulatory control of the CNS, the ENS can also function independently, a process involving interactions between enteric glial cells and immune regulation (Dora et al., 2021).

At the dawn of the 21st century, propelled by technological advancements in computational power and data processing capabilities, research in the field of metabolomics has accelerated substantially. These advancements in metabolomics have significantly enhanced our understanding of the gut microbiota, thereby enriching the landscape of gastrointestinal microbiome studies. It has been discovered that the activation of Toll-like receptors by lipopolysaccharides (LPS) plays an important role in the nervous system (Araldi et al., 2023), involving neuroimmune responses and the genesis of pain. Derived from gut Gram-negative bacteria, LPS gains access to the spinal cord through the bloodstream (Shen et al., 2017), subsequently activating Toll-like receptors (TLR) to partake in NP processing (Lin et al., 2022). Additionally, short-chain fatty acids (SCFAs), metabolic byproducts of the gut microbiota, play a critical role in microglial development (Erny et al., 2015). Multiple studies have demonstrated the key contributions of sphingolipids and polysaccharides produced by fragile rod-shaped bacteria in processes such as myelin formation, neuroinflammation, and the development of chronic pain (Mao et al., 2013; An et al., 2014; Joscelyn and Kasper, 2014; Blaho et al., 2015). These findings affirm the close connection between the CNS, microglia, and the gut microbiota.

At the same time, regarding tryptophan metabolism direction. Research evidence indicates that serotonin and quinolinic acid affect mood via the tryptophan metabolic pathway. In instances of intestinal inflammation, increased levels of quinolinic acid may contribute to anxiety (Kelly et al., 2016). Another crucial compound produced by a healthy gut microbiota is glutamate, which constitutes a vital link in the gut-brain axis (Zhang et al., 2019a). Mice subjected to acute stress-induced dysbiosis of the gut microbiota (due to antibiotic (ABX) treatment), exhibit a notable reduction in hippocampal N-Methyl-D-Aspartate (NMDA) and Brain-Derived neurotrophic factor (BDNF) levels, which can be restored by gut microbiota supplementation (Sudo et al., 2004; Gronier et al., 2018; Jiang et al., 2019). Furthermore, the gut microbiota directly engages in the regulation of certain neurotransmitters, such as GABA (Strandwitz et al., 2019) and 5-hydroxytryptamine (5-HT) (Yano et al., 2015), thereby influencing neuronal function. These findings greatly expand our understanding of the gut-brain axis.

### 2.2 The gut microbiota as a therapeutic target in neuropathic pain

Changes in gut microbiota were discernible in experiments employing various NP animal models. In an experimental model of NP induced by peripheral nerve injury, rats exhibiting dysbiosis in their gut microbiota were observed to be more susceptible to the development of NP (Yang et al., 2019). In the chronic sciatic nerve compression injury model, the abundances of Helicobacter, Phascolarctobacterium, Christensenella, Blautia, Streptococcus, Rothia and Lactobacillus were significantly increased, whereas the abundances of Ignatzschineria, Butyricimonas, Escherichia and Corynebacterium were significantly decreased (Chen et al., 2021). Additionally, experimental data indicate alterations in gut microbiota in C57BL/6 mice which are sensitive to Chemotherapy-induced peripheral neuropathy (CIPN). The abundance of bacteria *Akkermansia muciniphila* is significantly decreased, which may damage the integrity of the intestinal barrier, exposing the gut to bacterial metabolites, ultimately leading to brain dysfunction through the gut-brain axis (Ramakrishna et al., 2019). These significant changes in the gut microbiota imply a robust correlation between the gut microbiota and NP.

In fact, the critical role of gut microbiota in NP has been extensively reported. In animal experiments, researchers have demonstrated that the gut microbiota can to mitigate NP induced by chronic-constriction injury of the sciatic nerve (Ding et al., 2021). Furthermore, in the oxaliplatin (OXA) mice model, gut microbiota depletion effectively inhibits glial cell proliferation and cytokine production in the DRG induced by chronic constriction injury (CCI) or streptozotocin (STZ). The opposite is the partial recovery of the gut microbiota, especially Akkermansia and Bifidobacterium, which can either worsen or alleviate NP (Ma et al., 2022). CIPN is a prevalent adverse effect of cancer treatment. A sensitive neuronal *in vitro* model experiment demonstrated that gut probiotics hold promise for treating paclitaxel-induced NP, offering a long-term safe and effective treatment option (Castelli et al., 2018). Intriguingly, gut microbiota may not only contribute to the management of cancer-related NP but also potentially influence the effects of chemotherapy on tumors (Castelli et al., 2018; Vázquez-Baeza et al., 2018). These foundational experimental findings suggest that gut microbiota could represent a potential therapeutic target in CIPN.

Regarding the therapeutic role of the gut microbiome in NP, its underlying mechanisms have also garnered considerable attention and discussion. This intricate mechanism involves factors such as the metabolic products of the intestinal flora, inflammatory responses, and the recently focused neuroimmune responses. Apart from the involvement of gut microbiota, SCFAs, among the main metabolites produced by gut microbiota, have been implicated in NP. Depletion of SCFAs has been observed to alleviate NP induced by CCI (Zhou et al., 2021). Similarly, in the CCI model, following correlation analysis, it was revealed that the decreased abundance of gut microbiota producing butyrate is closely associated with levels of β-hydroxybutyric acid in both serum and spinal cord (Chen et al., 2021). This suggests that therapeutically targeting gut microbiota responsible for SCFA/β-hydroxybutyric acid production may holdpromise in ameliorating NP. Moreover, in examining the mechanism underlying inflammatory response, our investigation reveals that in the CIPN model, the therapeutic effect of probiotics on NP may be attributed to inflammation-related responses associated with the Interleukin (IL)-8 signaling pathway (Castelli et al., 2018). Conversely, modifying the gut microbiota composition through ABX treatment suppresses the TLR-4 signaling pathway mediated by macrophages, leading to improvements in CIPN and reduced secretion of inflammatory factors in the DRG (Lian et al., 2021; Ma et al., 2022). Additionally, a study revealed a significant proliferation of spinal cord microgliosis in mice receiving paclitaxel treatment, indicating a causal relationship. Gut microbiota plays a role in the development of CIPN by modulating microgliosis (Ramakrishna et al., 2019). This offers a promising avenue for the precise manipulation of gut microbiota in future NP management.

**Figure 2:**
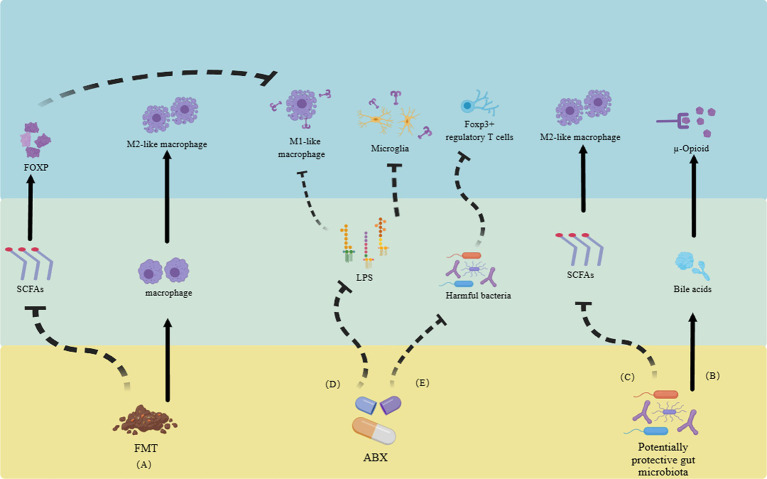
Utilizing gut microbiota and their metabolites to alleviate neuropathic pain through the neuroimmune system. **(A)** FMT reduces SCFAs, inhibiting the transformation of M1-like macrophages and promoting the increase of M2-like macrophages, ultimately relieving neuropathic pain. **(B)** Gut microbiota itself can promote the generation of bile acids, activate opioid receptors, and alleviate pain. **(C)** Some gut microbiota can increase the number of M2-like macrophages by inhibiting the production of SCFAs. **(D)**. ABX alleviate pain by reducing the activation of Toll-like receptors in LPS-mediated M1-like macrophages and microglia. **(E)** ABX can also improve pain by altering gut microbiota and regulating regulatory T cells. FMT, fecal microbiota transplantation. SCFAs, short-chain fatty acids. ABX, antibiotics. LPS, lipopolysaccharides (Reichenberger et al., 2013; Van Laar et al., 2019; Bonomo et al., 2020; Cuozzo et al., 2021; Fonseca-Rodrigues et al., 2021; Jing et al., 2021; Lian et al., 2021; Wang et al., 2021; Ma et al., 2022).

Recent studies on NP found that the gut microbiota actively participates in the onset and progression of pain by interacting with the neuroimmune system. In a mouse model of CCI, the alteration of gut microbiota via ABX led to an attenuation of NP induced by CCI. This phenomenon may be attributed to the modulation of the balance between pro-inflammatory and anti-inflammatory T cells (Ding et al., 2021). In terms of utilizing the gut microbiota for treatment (Figure 2; Table 1): (a) It has been confirmed that judicious supplementation of probiotics holds promise for pain management. In a pain model induced by lumbar disk herniation, supplementation with *Lactobacillus paracasei* significantly alleviated pain and mitigated inflammatory responses (Wang et al., 2021). (b). Fecal microbiota transplantation (FMT) is a common strategy in gut microbiota research. In a mouse model of NP triggered by obesity, FMT from lean mice effectively alleviated pain. One potential mechanism underlying this effect could be the reduction of RYR2-dependent calcium release in DRG neurons of obese mice following FMT (Bonomo et al., 2020). Additionally, M2-like macrophages play a pivotal role in FMT treatment for obesity-induced peripheral NP, actively participating in the pain relief process and displaying a high association with SCFAs (Bonomo et al., 2020). In mouse model of spinal cord injury, significant enrichment of the Firmicutes phylum was observed following FMT, ultimately promoting functional recovery (Jing et al., 2021). In a clinical study, alterations in the composition of gut microbiota were confirmed in patients with complex regional pain syndrome, characterized by a decrease in diversity and similar shifts in the Firmicutes and Bacteroidetes phyla as observed in animal models (Reichenberger et al., 2013). (c). ABX treatment presents another common approach for investigating gut microbiota-directed therapies. It has been demonstrated that the elimination of gut microbiota through ABX can inhibit the production of IL-6 in the DRG and improve NP (Ma et al., 2022). Additionally, ABX treatment can also alleviate CIPN through the macrophage pathway (Lian et al., 2021). In addition, clinical evidence suggests that chronic NP is often associated with cognitive impairments (Fonseca-Rodrigues et al., 2021). Supplementation with probiotics or FMT has shown potential in mitigating the harm caused by chronic NP and its associated cognitive impairment (Van Laar et al., 2019; Bonomo et al., 2020; Cuozzo et al., 2021). Unfortunately, due to ethical considerations, there is currently limited research on the direct therapeutic effects of gut microbiota in the treatment of NP.

**Table 1:** Probiotics treatment.

Probiotics	Function
Probiotics VSL#3 (*Lactobacillus acidophilus*, *Bacillus subtilis*, plant-derived Lactobacillus, *Escherichia coli*)	Effective relief of morphine-induced opioid tolerance in mice (Zhang et al., 2019a)
*Bifidobacterium bifidum*	Maintain intestinal homeostasis (Wang et al., 2014)
*Lactobacillus reuteri* NK33 and *Bifidobacterium adolescentis* NK98, *Lactobacillus helveticus* R0052 and *Bifidobacterium longum* R0175	It alleviates the depressive state during pain treatment through neuroimmune mechanisms (Messaoudi et al., 2011; Jang et al., 2019; Yang et al., 2024)
*Lactobacillus paracasei* S16	It noticeably relieves pain and inflammatory responses in patients with lumbar disk herniation-related neuralgia (Wang et al., 2021)
Probiotic DSF (*Lactobacillus plantarum*, *Streptococcus thermophilus*, *Bifidobacterium breve*, *Lactobacillus paracasei*, *Lactobacillus acidophilus*, *Bifidobacterium longum*)	*In vitro* experiments have demonstrated that probiotics rich in these bacterial communities can treat neuropathic pain caused by chemotherapy drugs and alleviate inflammatory responses (Castelli et al., 2018)

In summary, accumulating preclinical evidence suggests an important role of the gut microbiota in NP, involving complex mechanisms such as gut microbiota composition and metabolites, related immune responses, and activation of the gut-brain axis. We look forward to obtaining more relevant findings in clinical studies.

## 3 Gut microbiome and opioids in neuropathic pain

### 3.1 Opioid-induced gut microbiota dysbiosis

**Figure 3:**
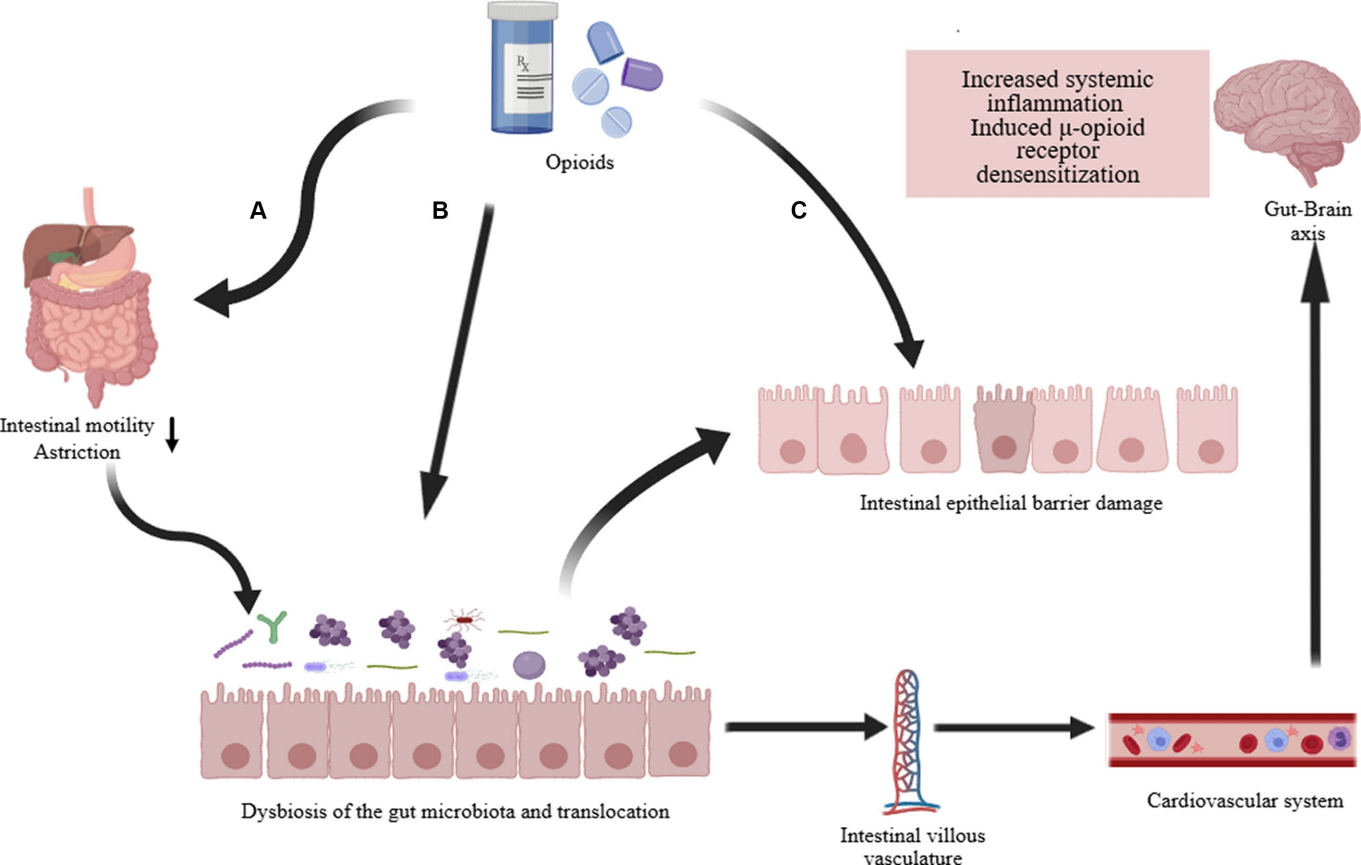
Opioid-induced gut microbiota dysbiosis. The long-term effects of opioid treatment on the intestines are: **(A)** Directly weakening gastrointestinal motility, commonly resulting in complications such as constipation. Changes in intestinal motility and transit time further alter the composition of the intestinal microbiota and cause translocation of gut microbiota. **(B)** Directly altering the composition and dysbiosis of the intestinal microbiota, such as a decrease in Lactobacillus and Bifidobacterium, an increase in Staphylococcus and Enterobacter, and potentially increasing other pathogenic bacteria. **(C)** Damage to the intestinal epithelial barrier (increased permeability, disruption of tight junction proteins, decreased immune function). The dysregulation of intestinal microbiota and function ultimately affects systemic inflammation and contributes to the development of opioid resistance via the gut-brain axis (Acharya et al., 2017; Bhave et al., 2017; Guo et al., 2019; Zhang et al., 2019a).

Opioids primarily exert their effects through G protein-coupled receptors, inhibiting the transmission of nociceptive signals from the periphery to the CNS (Zádori et al., 2023). Research indicates a close correlation between the use of opioids and the composition and function of the gut microbiota (Acharya et al., 2017; Guo et al., 2019). Prolonged opioid therapy can lead to a systemic inflammatory environment, evidenced in the gut microbiota by compromised barrier function, bacterial translocation, release of gut inflammatory factors, and disrupted gastrointestinal motility (Figure 3) (Zhang et al., 2019a). Recent studies report that prolonged morphine therapy can lead to the onset of intestinal inflammation, with its mechanism closely related to the bacterial product LPS (Bhave et al., 2017). Morphine treatment directly leads to increased activity of P2X receptors in enteric glia, ultimately triggering enhanced inflammatory responses (Bhave et al., 2017). An earlier report mentioned that Systemic administration of morphine can activate a glial -mediated inflammatory cascade in the spinal dorsal horn (Raghavendra et al., 2004). Additionally, morphine sulfate attenuate the reward behavior associated with dopamine due to the activation of the BDNF signaling pathway (Taylor et al., 2016). This leads to the activation of glial cells and impaired reward mechanisms, ultimately reshaping the composition of the gut microbiota through the gut-brain axis (Zhang et al., 2019a). Additionally, opioid medications (morphine) can induce gastrointestinal motility disorders such as constipation, primarily attributed to alterations in the gut microbiota (Touw et al., 2017). In summary, opioids affect the gut microbiome through intricate mechanisms, predominantly inducing the change that typically manifests unfavorably.

#### 3.1.1 The composition of gut microbiota

Intestinal motility and transit time are the primary determinants of gut microbiota composition (Zádori et al., 2023). Constipation induced by opioid therapy and compromised intestinal motility exerts a profound influence on the composition of the gut microbiome. In a mouse model of long-term morphine treatment, dysbiosis of the gut microbiota was observed (Table 2). This dysbiosis comprised an increase in the abundance of Staphylococcus, Enterococcus, and Proteus, alongside a decrease in the abundance of Bacteroidales, Clostridiales, and Lactobacillales (Akbarali and Dewey, 2017; Kim et al., 2021). Furthermore, alterations in gut microbiota composition also extend to other physiological functions, including the inhibition of bile acid secretion (Hu et al., 2022), suppression of bicarbonate secretion (Xu et al., 2022) and the inhibition of immune surveillance (Brosnahan et al., 2014). Similar studies have also highlighted that morphine enhances the toxicity of *Pseudomonas aeruginosa* (Babrowski et al., 2012). Representative Gram-negative bacteria in the gut microbiota, such as those belonging to the genera Bacteroides, play an important role in ameliorating gut inflammation and maintaining the homeostasis of intestinal epithelial cells through the production of SCFAs (Smith et al., 2013). Prolonged treatment with morphine can lead to a reduction in Bacteroidetes, consequently giving rise to intestinal inflammation and compromising the integrity of the intestinal epithelial barrier (Kang et al., 2017). Another illustrative example lies in the Gram-positive Firmicutes phylum (Lactobacillales), which mitigates intestinal inflammation through butyrate salts and regulatory T cells (Furusawa et al., 2013). Prolonged morphine treatment also leads to a significant reduction in the abundance of the Firmicutes phylum (Kang et al., 2017). Interestingly, naloxone (a classic antagonist of opioid stimulants) can effectively alleviate the reduction of thick-walled microbial organisms caused by prolonged morphine treatment (Meng et al., 2015). In another study, naloxone was also shown to normalize morphine treatment-induced gut dysbiosis (Wang et al., 2018; Essmat et al., 2023). This could mean that the dysregulation of gut microbiota caused by opioid drugs can potentially be alleviated through the judicious use of peripheral μ-opioid receptor antagonists, thereby reducing the side effects associated with opioid medications.

**Table 2:** Changes in gut microbiota diversity with opioid treatment regimen (animal studies).

Opioid	Dose, dosing regimen	Finding	References
Morphine	25 mg pellet (4 days)	↑Staphylococcus, Enterococcus	Meng et al. (2015)
	25 mg pellet (5–7 days)	↑Enterococcaceae, Staphylococcaceae, Bacillaceae, Streptococcaceae, Erysipelotrichaceae ↓Bacteroidetes, Proteobacteria, Actinobacteria, Acidobacteria, Actinobacteria, Acidobacte	Banerjee et al. (2016)
	10 mg/kg i.p. (4 days)	↑Ruminococcus ↓Lactobacillus	Lee et al. (2018)
	25 mg pellet (3 days)	↑Flavobacterium, Enterococcus, Fusobacterium	Wang et al. (2018)
	75 mg pellet (5 days)	↑Enterobacteriales ↓Bacteroidales, Clostridiales,	Kang et al. (2017)
	5–40 mg pellet (8 days)	↑Allobaculum, Peptostreptococcaceae ↓Lactobacillus, Bifidobacterium	Zhang et al. (2019a)
	10 mg/kg i.p. (12 days)	↑Verrucomicrobia ↓Bacteroides	Zhang et al. (2021)
Oxycodone	2 mg s.c (5 days)	↑Firmicutes ↓Bacteroidetes	Simpson et al. (2020)
Hydromorphone	7.5 mg pellet (8 days)	↑Bacteroides, Enterococcus ↓Lactobacillus, Lachnospiraceae	Sharma et al. (2020)

Recent research has revealed that morphine treatment induces an enrichment of bacteria producing β-glucuronidase in the mouse gut. This leads to the accumulation of chemotherapy drugs in the intestine, culminating in the onset of gut dysbiosis (Meng et al., 2023). For cancer patients undergoing long-term opioid treatment for pain management, the development of interventional drugs targeting the gut microbiota associated with this treatment could potentially mitigate the adverse effects of chemotherapy. In addition, an elevated abundance of Enterobacteriaceae is frequently considered as an indicator of gut dysbiosis (Rizzatti et al., 2017). Recent studies have confirmed that Enterobacteriaceae are markedly enriched in histological samples of inflammatory bowel disease (Gevers et al., 2014). Prolonged exposure to morphine results in a significant increase in the abundance of Enterobacteriaceae (Kang et al., 2017). Furthermore, disparities may exist in secondary intestinal microbiota, such as filamentous bacteria (Earley et al., 2023). Additionally, there have been reports suggesting that the presence of certain less abundant genera, such as Verrucomicrobia and the genus Akkermansia, may also be augmented under the influence of opioid analgesics (Kim et al., 2021; Abu et al., 2022).

The results of animal experiments regarding changes in gut microbiota species due to opioid use are robust and compelling. However, two remaining questions remain concerning the translatability of these findings to humans. First, the use of opioids in animal studies involves high doses and short treatment durations. Second, there is a notable discrepancy in the presence of 85% of secondary phyla between the murine gastrointestinal tract used for preclinical experiments and the human body (Hu et al., 2024). Whether these subtle differences will influence the outcomes of clinical experiments remains to be investigated. Encouragingly, although the data are limited, there have been reports in human studies that parallel those from animal research. Notably, these include a decrease in Ruminococcaceae (Acharya et al., 2017) and an increase in Enterobacteriaceae (Gicquelais et al., 2020).

#### 3.1.2 Translocation and immunomodulation

In recent years, there has been growing interest concerning the mechanism by which opioid drugs influence the gut microbiota. The latest data has forged a connection between opioid treatment and gut microbiota translocation. Several preclinical studies have corroborated that classical opioid drugs, including morphine (Meng et al., 2015; Wang et al., 2018; Zhang et al., 2019a) and hydromorphone (Sharma et al., 2020), can disrupt intestinal epithelial integrity and barrier function in mice. This disruption ultimately leads to bacterial translocation and gut inflammation. On one hand, opioids impede gastrointestinal motility and substance transport by binding to the opioid mu-receptors in the myenteric plexus of the gut (Goudsward et al., 2024), This impairment in bowel motility can lead to the overgrowth of harmful gut bacteria, potentially resulting in gram-negative sepsis (Meng et al., 2013). On the other hand, in a mouse model of sepsis, it has been shown that morphine treatment induces the dissemination of Gram-positive bacteria (Meng et al., 2015). The translocation of Gram-positive bacteria subsequently triggers an excessive expression of IL-17A and IL-6, ultimately compromis of intestinal epithelial barrier function and further exacerbating systemic inflammation (Meng et al., 2015; Kang et al., 2017). Similar conclusions have also been drawn from clinical data (Meng et al., 2015).

The displacement of gut microbiota induced by opioid medications can lead to disruptions in intestinal epithelial integrity (Banerjee et al., 2016) and trigger inflammatory immune responses (Thomas et al., 2022). We posit that future research might delve into the intricate interplay between gut microbiota and immune responses within the intestinal epithelial barrier. In animal models, it has been found that gut microbiota serves as a key determinant in initiating autoimmune T-cell responses (Berer et al., 2017). The gut microbiota is likely to influence the function of many innate immune cells, thereby actively participating in the regulation of immune responses (Santa-Cecília et al., 2019). Notably, research has demonstrated that morphine exhibits no discernible impact on the gut microbiota of mice with TLR2 knockout or immunodeficiency (Banerjee et al., 2016). This observation underscores the critical role of immune function in maintaining intestinal microbial homeostasis and preventing dysbiosis.

Opioids often exert a dual effect on immune regulation. They typically suppress the immune response of T cells and B cells (Roy et al., 2011). However, in the gastrointestinal tract, they can paradoxically activate the immune system, giving rise to inflammatory responses (Meng et al., 2013; Kang et al., 2017). Research has shed light on the key role of neuroimmune effects induced by opioids in the gastrointestinal tract. Morphine directly induce changes in intestinal epithelial function and compromise the integrity of tight junction proteins through TLR regulation (Banerjee et al., 2016). Evidence suggests that these neuroimmune effects actively contribute to maintaining intestinal epithelial barrier function (Talbot et al., 2020). However, prolonged opioid therapy inhibits immune responses (Banerjee et al., 2016), ultimately leading to dysbiosis and an altered composition of the gut microbiota. This, in turn, leads to the disruption of the intestinal epithelial immune barrier and the development of analgesic tolerance, presenting a significant challenge in opioid management.

### 3.2 The benefits of gut microbiota in opioid treatment

#### 3.2.1 Analgesic tolerance

Opioids represent the most commonly prescribed medications for pain management. However, their prolonged usage carries inherent risks including the development of analgesic tolerance, potential overdose, and even mortality. In cases of NP, the downregulation or decreased sensitivity of opioid receptors frequently leads to diminished effectiveness of opioids, resulting in opioid tolerance (Balogh et al., 2019; Li et al., 2023). The prevailing view posits that alterations in opioid receptors are the primary drivers of opioid analgesic tolerance. These changes encompass a variety of mechanisms, such as the downregulation of spinal opioid receptors (Balogh et al., 2019), desensitization stemming from adaptive modifications within opioid receptor cells (Bai et al., 2020), and the functional modulation of cell membranes resulting from the repeated use of opioid analgesics (Kliewer et al., 2019). In addition, the mechanism of opioid-induced tolerance to pain relief is a topic of ongoing debate and investigation. Several potential mechanisms have emerged from relevant studies, including immune activation and neuroinflammation (Deleo et al., 2004; Hutchinson et al., 2011; Wang et al., 2012), receptor desensitization and downregulation (Balogh et al., 2019), upregulation of protein kinase A, and the release of norepinephrine and acetylcholine (Ma et al., 2001). Recent research has highlighted the significant role played by gut microbiota dysbiosis in pain modulation and tolerance through the activation of the gut-brain axis. Changes in the composition of gut microbiota have been shown to directly impact opioid tolerance in mouse experiments (Kang et al., 2017). Notably, the use of vancomycin alone, resulting in a reduction of gram-positive bacteria, prevented the development of opioid tolerance (Kang et al., 2017). While prior research on opioid-induced analgesic tolerance primarily focused on spinal mechanisms (Porreca et al., 1998), recent years have witnessed a growing emphasis on peripheral dysregulation mechanisms (Rivat, 2016; Zhang et al., 2016). Studies have revealed the pivotal role of the dorsal DRG in downregulating mu-opioid receptor (MOR) gene expression in the spinal dorsal horn, consequently diminishing the analgesic efficacy of opioids such as morphine (Zhang et al., 2016). The gut microbiota primarily contributes to the onset and progression of pain through its interaction with DRG and nerve endings within the intestine (Xu et al., 2022). Furthermore, recent research underscores the importance of the DRG in the development of pain tolerance (Jin et al., 2023). Another study has revealed that the gut microbiota is involved in the development of morphine tolerance in primary afferent neurons through its interaction with tetrodotoxin-resistant (TTX-R) sodium channels (Mischel et al., 2018). These research findings confirm the important role of gut microbiota in the peripheral analgesic mechanism of opioids.

Furthermore, a substantial body of evidence supports the key role of neuroinflammation in the development of morphine tolerance (Trang et al., 2015; Gei et al., 2024; Wang et al., 2024). Within neuroinflammatory responses, microglia have long been recognized as important markers for observation. Laboratory findings indicate that the elimination of microglia can effectively prevent opioid-induced pain hypersensitivity (Burma et al., 2017). In addition to its role in the analgesic tolerance process, glial activation actively participates in the development of NP (Gei et al., 2024) and is a key element in the mechanisms related to opioid treatment (Martínez-Navarro et al., 2019). MOR stimulation can lead to the activation of microglia (Ferrini et al., 2013). Interestingly, this opioid-induced glial response may counteract some of the analgesic effects (Martínez-Navarro et al., 2019). Furthermore, specific metabolic products of gut microbiota, such as lipoteichoic acid and LPS, can also induce the activation of glial cells within the nervous system (Obata and Pachnis, 2016; Bhave et al., 2017). Once activated, glial cells release an array of cytokines into the internal environment (Leduc-Pessah et al., 2017), consequently facilitating the development of analgesic tolerance (Drossman and Szigethy, 2014). These reports suggest that reducing harmful gut bacteria, which are associated with increased pain tolerance, can effectively alleviate the development of pain tolerance.

Recent studies have indicated that a portion of morphine analgesic tolerance arises from a decrease in the levels of intestinal β-glucuronidase, resulting in reduced hydrolysis of morphine metabolites back into morphine (Wang and Roy, 2016). Compelling evidence indicates that specific bacteria in the gut, such as Bifidobacteria and Lactobacilli, produce β-glucuronidase (Zhang et al., 2019a). Moreover, further research has confirmed the association of Bifidobacterium and Lactobacillus with opioid tolerance. Probiotics enriched with these microbial strains have demonstrated their effectiveness in alleviating analgesic tolerance in morphine-treated mice (Zhang et al., 2019a). The observed changes in Bifidobacterium and Lactobacillus are particularly intriguing, as these strains themselves contribute to maintaining intestinal homeostasis (Wang et al., 2014). Furthermore, recent investigations into opioids have revealed that FMT significantly reduces opioid withdrawal responses (Zhang et al., 2021). In summary, these studies underscore the substantial role of gut microbiota in opioid treatment, suggesting that supplementation with associated gut microbial strains may potentially prolong the analgesic efficacy of opioids.

#### 3.2.2 The addictive properties of opioids

Opioid addiction poses a significant challenge in the context of long-term opioid use for NP, often leading to varying degrees of withdrawal symptoms upon discontinuation of the medication (Simpson et al., 2020). Preclinical evidence strongly suggests that the gut microbiota actively influences behaviors associated with opioid addiction (Han et al., 2018; Lee et al., 2018; Hofford et al., 2021; Thomaz et al., 2021). Recent research has been exploring the impact of gut microbiota on opioid withdrawal. A recent preclinical study yielded results supporting the effectiveness of FMT and ABX treatment in reducing naloxone-induced withdrawal responses (Thomaz et al., 2021). Furthermore, a study identified significant gender differences in the relationship between gut microbiota and opioid addiction (Ren and Lotfipour, 2023). Among the abundant evidence, a key produced by the gut microbiota, SCFAs, plays a crucial role in cocaine reward behavior (Kiraly et al., 2016). In instances where gut microbiota diversity is inhibited by ABX, there is an increase in cocaine-conditioned place preference (CPP), a phenomenon that can be reversed through SCFA supplementation (Kiraly et al., 2016; Hofford et al., 2021). This suggests that diminished gut microbiota diversity, likely closely linked to SCFAs, instigates changes in opioid reward behavior. Research on SCFAs holds promise for the development of safer and more effective opioid treatment options.

Chronic morphine treatment activate the BDNF pathway through microglia, resulting in a reduction of dopamine-dependent reward behavior (Taylor et al., 2016). This underscores microglia as a key factor in the development of reward dysfunction induced by opioid drugs. Excessive activation of microglia in the ventral tegmental area leads to impaired brain reward behavior and diminished pleasure (Taylor et al., 2016), necessitating higher opioid doses for achieving reward and ultimately contributing to opioid addiction. Importantly, the gut microbiota can influence the morphology and activity of microglia (Gicquelais et al., 2020), consequently leading to reduced dopamine activity and disruptions in reward mechanisms (Taylor et al., 2016). Recent animal experiments have demonstrated that animals treated with FMT exhibited a significant restoration of normal reward behavior, accompanied by an observed increase in resting-state microglia (Lee et al., 2018). These research findings suggest that modulating the gut microbiota can exert a positive influence on the restoration of reward behavior and microglial function. Advancements in gut-brain axis research have suggested intriguing connections between gut microbiota, neuroimmune responses, and opioid withdrawal, offering fresh insights into opioid pain management. The development of gut microbiota-based therapies has introduced a fresh perspective to opioid withdrawal treatment and neuroimmune mechanisms. However, extensive clinical research is required to conclusively validate these possibilities and to further investigate the interactions between gut microbiota, opioid management, and neuroimmunity.

#### 3.2.3 Mental Health

NP is often accompanied by a spectrum of depressive symptoms (Janakiram et al., 2023). Notably, the severity of depression tends to worsen in tandem with the intensity of pain (Agüera-Ortiz et al., 2011). Clinical psychologists often use sedative medication as an adjunct treatment during the process of treating depression. When considering the combination of opioid drugs and sedative drugs, particularly benzodiazepines, it is imperative to exercise a high degree of vigilance due to the potential risks associated with these medications (Liu et al., 2021). Previous studies have confirmed that when compared to using opioids in isolation for managing NP, the combination of opioids and sedative drugs may lead to a notable increase in the risk of drug overdose (Meyer et al., 2023). Furthermore, it can escalate to a critical level, potentially culminating in fatal respiratory depression (Liu et al., 2021). Research has shown that the activation of opioid receptors in the context of pain treatment can lead to or exacerbate depressive symptoms (Dagher et al., 2022). This pertains not only to patients already affected by mental health issues, but even to those deemed mentally healthy, long-term use of opioids significantly increases the risk of developing depression (Scherrer et al., 2016).

The gut microbiota plays a pivotal role in the development of depression by secreting neurotransmitters, including serotonin (Yano et al., 2015). The utilization of opioid medications can disrupt the gut microbiota, resulting in a depletion of serotonin levels and an augmented risk of developing depression (Parker et al., 2020). Notably, Lactobacillus and Bifidobacterium have been implicated in associations with depression and anxiety (Han and Kim, 2019). Their depletion can occur as a consequence of morphine treatment (Zhang et al., 2019a), potentially increasing the susceptibility to experiencing anxiety and depression during medication therapy. Encouragingly, specific strains of probiotics, such as Bifidobacterium, have demonstrated the capacity to mitigate depression by increasing BDNF levels and reducing oxidative stress (Messaoudi et al., 2011). Research has reported a decrease in BDNF levels in instances of depression, and clinical studies have shown that supplementation with probiotics can lower depressive symptoms (Jang et al., 2019). Another study on Bifidobacterium revealed that it, along with Lactobacillus, contributes to the reduction of depression levels by activating the vagus nerve and eliciting an immunostress response (Yang et al., 2024). Furthermore, research findings also suggest that increasing the abundance of the Ruminococcaceae family could also be a potential avenue for reducing depression levels (Zhu et al., 2019). A recent study suggests that Lactobacillus may help alleviate depression and anxiety induced by chronic stress by sustaining levels of interferon gamma (IFN-γ). Therefore, maintaining healthy levels of Lactobacillus or IFN-γ could potentially prevent or treat symptoms of depression (Clark et al., 2023). Although further exploration is warranted to fully understand the connection between depression and the gut microbiota, it is worth considering supplementation with specific gut microbiota, especially in cases necessitating long-term opioid medication use for NP treatment.

A clinical study has revealed that patients with higher depression scores often exhibit elevated levels of kynurenine, serotonin, and histamine (Han et al., 2022). These compounds are primarily produced by enterochromaffin cells under the influence of gut microbiota, ultimately contributing to the onset of depression. This also underscores the indirect influence of gut microbiota on the development of depression. In addition, another article highlights the serotonin-generating capability of *Clostridium nexile*, which demonstrates significant elevation in patients with severe depression (Williams et al., 2014). These clinical findings suggest a potential avenue for alleviating the depressive effects induced by opioid treatment, by targeting specific gut microbiota, such as *Clostridium nexile*, to modulate neurotransmitter production.

## 4 Conclusions and treatment perspectives

With the rapid development of genomic sequencing technology, research on gut microbiota has entered a transformative phase (Jovel et al., 2016). These microorganisms, cohabiting with humans, have demonstrated significant influence over various aspects of our physiological and pathological behaviors (Barko et al., 2018; Pane et al., 2022). It is imperative that we focus on and deepen our understanding of gut microbiota and their metabolites within the human body, to gain insights into human health and address unresolved diseases. Research on the gut-brain axis has received considerable attention, with emerging evidence indicating that gut microbiota and their metabolites (SCFAs, LPS, butyrate, and glutamate) play pivotal roles in many diseases (Furusawa et al., 2013; Bhave et al., 2017; Zhang et al., 2019a), including NP. The mechanisms underlying these effects involve neuroimmune interactions. Extensive experimental data have demonstrated the intricate and complex connections between gut microbiota and the development of pain, including NP (Vázquez-Baeza et al., 2018; Ramakrishna et al., 2019; Lian et al., 2021; Ma et al., 2022). Probiotic supplementation or FMT has demonstrated significant reductions in NP (Vázquez-Baeza et al., 2018; Bonomo et al., 2020; Wang et al., 2021). Exploring the involvement of gut microbiota in the pathogenesis of NP holds promise for the development of novel therapeutic approaches in pain management for the future.

In traditional approaches to treating NP, opioids have held a pivotal role (Balanaser et al., 2023). However, their effectiveness is often limited due to shared neurophysiological mechanisms with NP (Martínez-Navarro et al., 2019), along with associated risks of tolerance, addiction, and potential overuse crises. This article elucidates the intricate interplay between gut microbiota and opioid drugs in the context of NP. Opioid therapy alters gut microbiota diversity (Kim et al., 2021) and physiological metabolic processes (Sharma et al., 2020; Kim et al., 2021) by affecting gastrointestinal motility and neuroimmune function. Simultaneously, interventions such as probiotics, ABX, and FMT significantly reduce opioid tolerance and addiction (Wang et al., 2014; Wang and Roy, 2016; Kim et al., 2021; Thomaz et al., 2021) while also positively affecting the psychological well-being of the host (Table 3) (Jang et al., 2019). These findings hold relevance in clinical practice, including observational studies, suggesting that gut microbiota can play a valuable role in the management of opioid-induced NP, thereby mitigating the adverse effects of opioids and ultimately enhancing the overall quality of treatment.

**Table 3:** The benefits of gut microbiota in the management of opioids (animal studies).

Side effects of opioids	Involvement of gut microbiota
Analgesic tolerance	A reduction in Gram-positive bacteria has led to a decrease in the activity of microglial cells (Obata and Pachnis, 2016; Bhave et al., 2017), which has alleviated opioid tolerance (Kang et al., 2017)
	The gut microbiota can actively participate in the development of opioid tolerance through the dorsal root ganglia (Corder et al., 2017)
	Bifidobacteria and Lactobacilli effectively increase the hydrolysis of opioid metabolites back to morphine through β-glucuronidase (Wang and Roy, 2016; Zhang et al., 2019b)
Addictive	FMT and ABX can reduce the withdrawal reactions induced by naloxone (Thomaz et al., 2021)
	In a FMT animal model, increased activity of microglia and restoration of normal opioid reward behavior were observed (Lee et al., 2018)
Mental health	Lactobacillus and Bifidobacterium alleviate depressive and anxiety behaviors through activation of the neuroimmune system (Clapp et al., 2019), but they can be depleted by opioid treatment (Zhang et al., 2019a)
	Lactobacillus relieves depression and anxiety by maintaining the level of compound interferon gamma (Clark et al., 2023)
	Opioid treatment leads to a decrease in serotonin levels, while certain gut microbiota can improve depressive behavior by secreting serotonin (Agüera-Ortiz et al., 2011; Janakiram et al., 2023)
	*Clostridium nexile* exacerbate the depressive state induced by opioids through the production of serotonin (Williams et al., 2014)

The nexus between gut microbiota, opioids, and NP presents both intriguing and challenging dimensions. Presently, most research is centered around animal models, underscoring the need for further clinical studies and long-term dynamic data to support the underlying mechanisms involving gut microbiota. Therefore, future clinical research must focus on generating a robust body of evidence and thoroughly investigating the relevant mechanisms to elucidate the complex relationship between gut microbiota and various types of NP. Furthermore, the influence of numerous confounding factors, including patients lifestyles and dietary habits, complex psychological factors, and diverse treatment regimens, must be carefully considered in studies exploring the interactions between advanced gut microbiota-dietary interventions and gut microbiota-based therapies. Additionally, advancements in detection methods are expected to unveil previously overlooked microorganisms, thereby prompting scientists to deepen their understanding of the interconnections among these variables. We look forward to the widespread integration of gut microbiota-based approaches in opioid management for NP in the future.

## Author contributions

ZG: Conceptualization, Supervision, Writing – original draft, Writing – review & editing. QX: Conceptualization, Data curation, Investigation, Writing – review & editing. YL: Investigation, Supervision, Validation, Writing – review & editing. BY: Methodology, Validation, Writing – review & editing. BH: Writing – review & editing, Supervision. ZL: Funding acquisition, Writing – original draft, Writing – review & editing.
